# Reference Guided De Novo Genome Assembly of Transformation Pliable *Solanum lycopersicum* cv. Pusa Ruby

**DOI:** 10.3390/genes14030570

**Published:** 2023-02-24

**Authors:** Sanskriti Vats, Virender Kumar, Rushil Mandlik, Gunvant Patil, Humira Sonah, Joy Roy, Tilak Raj Sharma, Rupesh Deshmukh

**Affiliations:** 1 National Agri-Food Biotechnology Institute (NABI), Mohali 140306, Punjab, India; 2 Regional Centre for Biotechnology, Faridabad 121001, Haryana, India; 3 Department of Biotechnology, Panjab University, Chandigarh 160014, Punjab, India; 4 Institute of Genomics for Crop Abiotic Stress Tolerance, Department of Plant and Soil Science, Texas Tech University, Lubbock, TX 79410, USA; 5 Department of Crop Science, Indian Council of Agriculture Research (ICAR), Krishi Bhavan, New Delhi 110001, Delhi, India; 6 Department of Biotechnology, Central University of Haryana, Mahendragarh 123031, Haryana, India

**Keywords:** whole genome sequencing, *Solanum lycopersicum*, tomato, Pusa Ruby, reference-guided assembly, transformation protocol, *Agrobacterium tumefaciens*

## Abstract

*Solanum lycopersicum* cv. Pusa Ruby (PR) is a superior tomato cultivar routinely used as a model tomato variety. Here, we report a reference-guided genome assembly for PR, covering 97.6% of the total single-copy genes in the solanales order. The PR genome contains 34,075 genes and 423,288 variants, out of which 127,131 are intragenic and 1232 are of high impact. The assembly was packaged according to PanSol guidelines (N50 = 60,396,827) with the largest scaffold measuring 85 megabases. The similarity of the PR genome assembly to Heinz1706, M82, and Fla.8924 was measured and the results suggest PR has the lowest affinity towards the hybrid Fla.8924. We then analyzed the regeneration efficiency of PR in comparison to another variety, Pusa Early Dwarf (PED). PR was found to have a high regeneration rate (45.51%) and therefore, we performed allele mining for genes associated with regeneration and found that only *AGAMOUS-LIKE15* has a null mutation. Further, allele mining for fruit quality-related genes was also executed. The PR genome has an *Ovate* mutation leading to round fruit shape, causing economically undesirable fruit cracking. This genomic data can be potentially used for large scale crop improvement programs as well as functional annotation studies.

## 1. Introduction

Tomatoes (*S. lycopersicum* L.) are an important horticultural vegetable fruit crop in the Solanaceae family which has acquired an important place in fruit biology [1,2]. Recently, tomatoes have also been established as a model plant for functional genomics due to their small diploid genome, short generation time, amenable transformation, and substantial genomic resources [3]. The resequencing of hundreds of tomato varieties was initiated soon after the [4] published the first reference genome of a tomato (Heinz 1706) and has increased the utility of tomato as a model crop. However, since its introduction from Europe to different parts of the world, the tomato has been subjected to ongoing selection resulting in reduced genetic variation [5]. Conventional breeding and induced mutations have helped in the introgression of beneficial traits from wild relatives. Whole genome sequencing data helps in the exploitation of natural variation for economically important traits [4,6].

**Figure 1:**
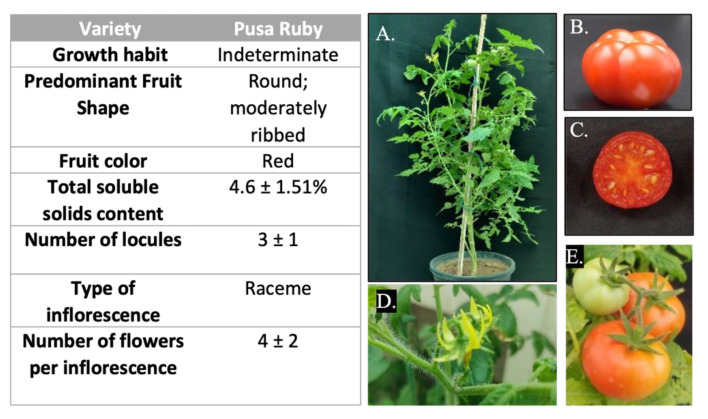
Morphological characteristics of *S. lycopersicum* cv. Pusa Ruby. The table on the left side describes the different phenotypic attributes of *S. lycopersicum* cv. Pusa Ruby. (**A**) Indeterminate growth habit of pusa ruby plants. (**B**,**C**) Red colored, and round fruits with slight ridges and 3–4 locules. (**D**) Raceme inflorescence is present in Pusa Ruby plants with non-synchronous fruit ripening, a characteristic of indeterminate plants. (**E**) Immature and ripening fruits on Pusa Ruby plant.

Continuous breeding and selection have led to a plethora of region-specific varieties with differing fruit size, shape, shelf life, nutritional quality, antioxidant capacity, plant growth habit, and other organoleptic properties [7,8,9,10,11]. One such variety is *S. lycopersicum* cv. Pusa Ruby (PR), which is particularly amenable to *Agrobacterium* mediated transformation and regeneration [12,13,14,15,16]. Pusa Ruby was released by IARI, New Delhi (year of notification 1973), and is a selection from the cross between Sioux and Improved Meeruti (https://www.iari.res.in/index.php?option=com_content&view=article&id=226&Itemid=696; accessed on 23 June 2022). It is an indeterminate, early growing variety with medium-sized, round, lobed, and slightly ribbed fruits, suitable for both table and processing purposes (Figure 1) (http://ztmbpd.iari.res.in/technologies/varietieshybrids/vegetables/tomato/; accessed on 17 June 2022). In India, the majority of biotechnological studies involving transformation are performed on PR [17,18,19,20,21,22,23,24,25]. Multiple functional annotation, phenotypic, and transcriptomic studies also use the PR genotype [26,27,28,29,30]. Therefore, a large amount of phenotypic data is also available for this variety which makes it suitable for whole genome resequencing analysis.

In the current study, we have generated a reference guided genome assembly for PR followed by the identification of variations, as well as structural and functional annotation. Furthermore, variation in the genes specific to fruit quality and transformation efficiency were also studied. Pusa Ruby is the predominant variety used in CRISPR/Cas-based studies because of its high regeneration efficiency, therefore, we have focused on its regeneration and transformation efficiency in comparison to another variety. Here, we present a simple method for the reference guided genome assembly and regeneration of transgenics in the PR variety.

## 2. Materials and Methods

### 2.1. Plant Materials and DNA Isolation

Pusa Ruby plants were grown in a greenhouse under a temperature of 24 °C and a 16/8 h day/night cycle. DNA was isolated from fresh young leaves using the CTAB method [31]. The DNA extracted was quantified on NanoQuant, Infinite M200 PRO (Tecan, Mannedorf, Switzerland) and analyzed for quality on 0.8% agarose gel.

### 2.2. The Whole Genome Paired-End Library Preparation and Sequencing

The whole genome sequencing library was prepared using 150 ng of intact DNA by QIAseq FX DNA Library Kit for Illumina (Catalog: 180479, QIAGEN, Hilden, Germany) using the manufacturer’s instructions. The library concentration was determined using Qubit.3 Fluorometer (Catalog: Q33216, Life technologies, Carlsbad, CA, USA) using The Qubit™ 1X dsDNA HS (High Sensitivity) Assay Kit (Catalog: Q32854, ThermoFisher Scientific, Waltham, MA USA). The Agilent D5000 ScreenTape System in a 4150 TapeStation System (Catalog: G2992AA, Agilent, Santa Clara, CA, USA), designed to analyze 35–1000 bp long sequences were used to validate the quality of the library. The sequencing was performed using Illumina NovaSeq 6000 system.

### 2.3. Quality Control and Read Processing

Read quality was analyzed using FastQC v0.11.9 [32] (https://www.bioinformatics.babraham.ac.uk/projects/fastqc/, accessed on 25 April 2022). The reads were trimmed for adapter contamination (Table S1) and further based on the QC analysis of raw reads, c15 bp forward and 4 bp reverse sequences were trimmed using CLC genomics workbench v21 release (https://digitalinsights.qiagen.com/products-overview/discovery-insights-portfolio/analysis-and-visualization/qiagen-clc-genomics-workbench/, accessed on 26 April 2022). The commands used in all the tools are given in File S1.

### 2.4. K-mer Analysis and Heterozygosity Estimation

The K-mer size was estimated using JELLYFISH tool (http://www.cs.cmu.edu/~ckingsf/software/jellyfish/, accessed 29 January 2023) [33]. The results from this tool were used to estimate genome heterozygosity for PR by GenomeScope ver. 1.0 (https://github.com/schatzlab/genomescope, accessed on 30 January 2023) [34].

### 2.5. Reference Guided De Novo Genome Assembly

#### 2.5.1. De Novo Genome Assembly

CLC workbench v21 and SOAPdenovo software/tool were used to generate de novo assembly from the paired end reads (http://soap.genomics.org.cn/soapdenovo.html, accessed on 27 April 2022) [35]. For CLC, the two reads were imported into CLC as customized illumina reads. The reads were then trimmed as mentioned above and checked for quality using CLC as well as FastQC [32]. The trimmed and error corrected reads were thereafter utilized for de novo genome assembly. Default parameters were used for CLC. Contigs shorter than 200 bp were discarded.

#### 2.5.2. Filtering Chloroplastic, Mitochondrial and rDNA Genes

Solanum chloroplast, mitochondrial and ribosomal DNA were downloaded from NCBI nucleotide database in bulk. The GenBank accession IDs for Solanum chloroplast, mitochondria, and rDNA sequences used in this study are given in Table S2 [36]. The makeblastdb (v2.5.0+) application was used to create local BLAST databases with chloroplast, mitochondrial, and rDNA sequences [37]. Blastn with output format 6 was used to align these sequences with PR de novo genome assembly. The blastn output was custom sorted according to e-value, query length, and query coverage in Microsoft excel version 2204 (2019). Then, contigs with query coverage of 60% or more with the rDNA database were marked as rDNA (2759 contigs). Contigs aligned to the chloroplast database with query coverage of 40% or more were deemed as chloroplast sequences (1072 contigs), and contigs that aligned with 50% query coverage with mitochondrial database were marked as putative mitochondrial contigs (1904 contigs). A total of 5142 contigs were removed from the de novo assembly before scaffolding.

#### 2.5.3. Scaffolding

The draft de novo genome assembly without putative chloroplast, mitochondrial, and rDNA sequences was ordered and oriented into scaffolds by RagTag using default parameters (https://github.com/malonge/RagTag/wiki/scaffold, accessed on 30 April 2022) based on *S. lycopersicum* reference genome ITAG 4.0. The reference assembly was downloaded from the solgenomics.net (https://solgenomics.net/ftp/tomato_genome/Heinz1706/assembly/build_4.00/, accessed on 22 April 2022). RagTag is a reference-based scaffolder that stitches individual contigs by inserting 100 Ns [38]. This tool generated 13 chromosome sequences (00 to 12) and another 96,619 unplaced contigs (total size: 35,061,362). 

### 2.6. The Repetitive Elements in the Sequence

Repetitive elements in the assembled genome were masked using RepeatMasker v4.1.2 [39]. Transposable elements in the PR genome were detected by The Extensive de novo TE Annotator (EDTA) v2.0.0 (https://github.com/oushujun/EDTA/blob/master/README.md, accessed on 15 May 2022) [40].

### 2.7. Assessing Assembly Completeness and Quality Control

The assembly completeness was evaluated by BUSCO (Benchmarking Universal Single-Copy Orthologs) software (version 5.3.2; https://busco.ezlab.org/, accessed on 20 May 2022) [41]. The solanales_odb10 database was used to find orthologous genes in genome mode and with MetaEuk gene predictor [42]. This tool identifies the presence of single copy orthologs to assess the completeness of the generated assembly.

### 2.8. Assembly Packaging

The assembly was packaged according to pansol guidelines. Chromosome names and unmapped contigs were named according to the guidelines by pansol and the chromosome names in ITAG4.0.

### 2.9. Structural and Functional Annotation of the Pusa Ruby Genome

For structural and functional annotation of the PR v1.0 assembly, we used liftoff using ITAG 4.0 gene models [43]. For ab initio gene prediction, AUGUSTUS v2.5.5 was used [44]. The annotation files generated were used as an input for gffread v.0.12.7 for extracting transcript, amino acid, and coding sequences [45]. For gene ontology (GO) enrichment analysis of the designated genes, we used gene ontology consortium, which is a panther-based online tool (http://geneontology.org/, accessed on 12 May 2022). Fisher’s exact test for calculating the false discovery rate was used by this tool to map the input gene IDs to the reference list.

### 2.10. Mapping of Illumina PE Short Reads to S. lycopersicum Assembly (Build 4.0)

The high-quality genomic paired end raw reads were also mapped to the *S. lycopersicum* reference genome assembly build 4.0 (https://solgenomics.net/ftp/tomato_genome/Heinz1706/assembly/build_4.00/, accessed on 22 April 2022) using BWA-MEM [46] and CLC genomics workbench [47]. The alignment files generated were processed using SAMtools.

### 2.11. Analyses of SNPs, InDels, and Structural Variants

The dnadiff tool from the MUMmer package resulted in an snp file, which was used to study the SNPs and InDels present in the PR genome. The snp file was converted into variant call format using all2vcf tool (https://github.com/MatteoSchiavinato/all2vcf, accessed on 22 April 2022). To verify the results, the CLC genomics workbench v21 release (www.qiagenbioinformatics.com/products/clc-genomics-workbench/, accessed on 22 April 2022) was also used to map the PE reads to the *S. lycopersicum* reference genome (SL 4.0). In addition, BWA-MEM was used to generate .bam file from which structural variants and SNPs were further elucidated using *S. lycopersicum* NCBI release 3.0 [48]. SNPEff tool was used to characterize the functional effect of variants (synonymous, non-synonymous, downstream variants, upstream variants, modifiers etc.) [49].

### 2.12. Whole Genome Comparison of Pusa Ruby against Heinz1706, M82, and Fla

The genome assemblies of *S. lycopersicum* varieties Heinz1706 (SL4.0 reference assembly), M82, and Fla were downloaded from https://solgenomics.net/. The delta file generated from the NUCmer tool of the MUMmer package v4.0.0rc1 was used as input for the dnadiff tool included in MUMmer package as well (https://github.com/mummer4/mummer/releases, accessed on 3 May 2022) [50]. The output of the dnadiff file contained statistics of the differences between the genome assemblies of PR and Heinz1706, M82, and Fla.

### 2.13. Identification of Orthologous Genes

To analyze the functional relatedness of predicted PR genes with other Solanaceae crops, clustering of orthologous proteins was performed with orthofinder v2.5.4 tool (https://github.com/davidemms/OrthoFinder/releases/download/2.5.4/OrthoFinder.tar.gz, accessed on 10 June 2022) [51]. The protein sequences of Solanaceae species (*Capsicum annum*, *S. lycopersicum*, *S. melongana*, *S. lycoeprsicum var. cerasiforme*, *S. pimpinellifolium* and *S. tuberosum*) were downloaded from https://solgenomics.net/ (accessed on 8 June 2022). *Arabidopsis* and rice were used as outgroups. The phylogenetic tree based on orthologous was visualised using dendroscope v3.8.3.0 [52].

### 2.14. Allele Mining of Putative Genes Involved in Regeneration and Fruit Quality

The tomato homologs for selected genes defined by BLASTX search against the reported genes. The genes with the least e-score and maximum percent similarity were selected as putative genes involved in the regeneration process and fruit quality.

### 2.15. Construct Preparation and Plant Transformation

The pMOD_B2103b, pMOD_C2906, and pMOD_A0501 vectors were used for construct preparation [53]. The sgRNAs were first cloned into pMOD_B2103b and further moved into T-DNA backbone along with Cas9. Ampicillin, kanamycin and ccdb were used for the selection of positively transformed *E. coli* DB3.1 colonies.

The final constructs were transformed into PR and Pusa Early Dwarf (PED) by *Agrobacterium tumefaciens* strain LBA4404 mediated stable transformation [54]. Seeds were sterilized with 70% ethanol for 1 min, followed by 3 min in between 20 and 15 min in sodium hypochlorite (4% *v*/*v*). Seeds were rinsed at least four times with distilled water before sowing in jam bottles with 4.4 g MS salts, 15 g sucrose, and 8% agar (pH 5.8). Cotyledons and hypocotyls were excised 8–10 days after sowing and put on pre-culture medium containing MS salts with 30 g/L sucrose, B5 vitamins, 2 mg/L zeatin, 0.1 mg/L indole acetic acid (IAA), 100 µM acetosyringone, and 8% agar (pH 5.8). The pre-culture plates were incubated for 24 h in dark growth chambers with 24 ± 2 °C. Meanwhile, *Agrobacterium* cultures were prepared in YEB medium with appropriate selection reagents. Liquid ½ MS medium (pH 5.8) was used to wash *Agrobacterium* cultures. The explants on pre-culture medium were treated with *A. tumefaciens* cultures for five minutes, dried on sterilized filter paper, and cultured for 72 h in the dark at 23 °C on co-culture medium with the same composition as pre-culture medium. Afterward, explants were washed with liquid MS medium, dried, and put on 1X selection medium (MS salts, B5 vitamins, 30 g/L sucrose, 2 mg/L zeatin, 0.1 mg/L indole acetic acid (IAA), 50 mg/L kanamycin, 350 mg/L timentin, 8% agar and pH 5.8). After two weeks, the explants were subcultured onto 2X selection medium with 100 mg/L kanamycin and the remainder of the composition same as 1X selection medium. The subculturing after every two weeks on 2X selection medium was continued until the appearance of shoots with at least one internode. The regenerated shoots with at least one internode were transferred to the rooting medium (MS salts with B5 vitamins, 30 g/L sucrose, 350 mg/L timentin, and 4% agar). Shoots with ample rooting were transferred into soilrite and acclimatized in growth chambers at 22 °C before shifting to greenhouse. PCR and kanamycin leaf assay were used for the transgenic identification. The primers used for transgene identification are provided in Table S3.

## 3. Results

### 3.1. K-mer and Genome Heterozygosity Estimation

**Figure 2:**
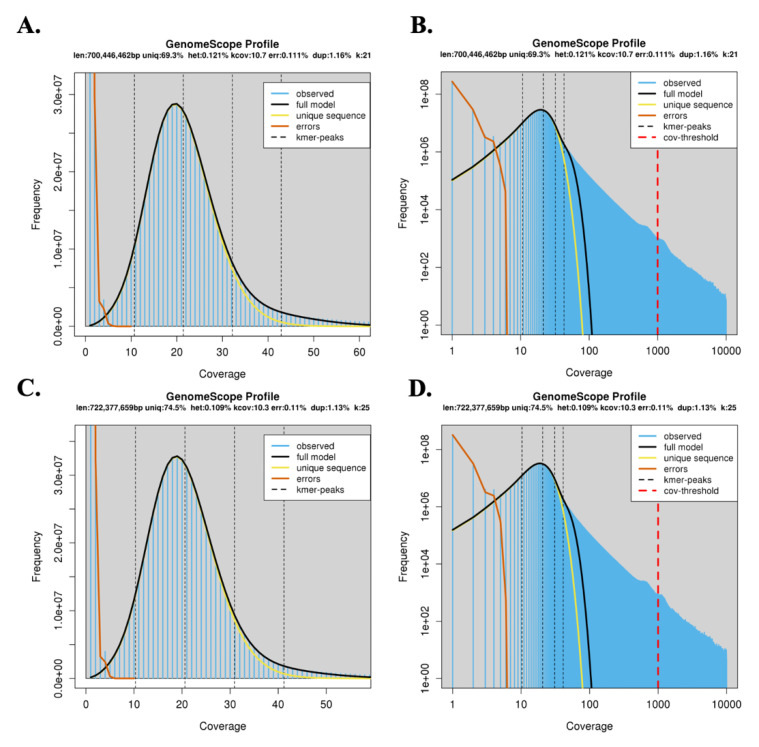
GenomeScope profile of the Pusa Ruby short reads using k-mer size 21 (**A**,**B**), and 25 (**C**,**D**). The larger peak is the homozygous region of the genome, accounting for unique 21-mers (**A**,**B**), and 25-mers (**C**,**D**) in both the strands of DNA. Low heterozygosity and repeat content were found using this analysis, with coverage being 10.3. Error in sequencing due to PCR is represented by the unique sequences on the left of the graph. The % PCR duplication was also found to be low.

The quality checked raw reads were used for the genome heterozygosity estimation here, using GenomeScope ver. 1.0 (http://qb.cshl.edu/genomescope/, accessed on 30 January 2023). The genome size and repeat content were also estimated from the processed raw reads using GenomeScope ver. 1.0. We used the default k-mer size 21-mer and 25-mer (Figure 2). The 21-mer size displayed model fitness 94.98–99.08% and read error rate of 0.112%, while 25-mer displayed model fit 95.95–99.107% and read error rate 0.109% (File S2a,b). Therefore, we used the 25-mer for further analysis. Based on the 25-mer analysis, the PR genome size was found to be around 723 Mb approximately, and genome heterozygosity was found to be 0.105–0.113%. The genome haploid length was found to be a maximum of 722,377,659 bp, of which 538,412,765 bp (74.54%) were found to be unique and 183,964,894 bp (25.46%) were repeated sequences (File S2b). Low heterozygosity and repeat content are desirable features for generating a reliable genome assembly using short read sequencing. Further, 3.0 × 10^7^ unique 25-mers were observed 20 times. Low number of unique K-mers with 1–7 frequency due to sequencing errors were observed. Together with these results, we could conclude that the PR genome sequenced is highly homozygous with low repeat content and can be effectively used to generate quality assembly.

### 3.2. Reference Guided Genome Assembly of S. lycopersicum cv. Pusa Ruby and Quality Assessment

**Figure 3:**
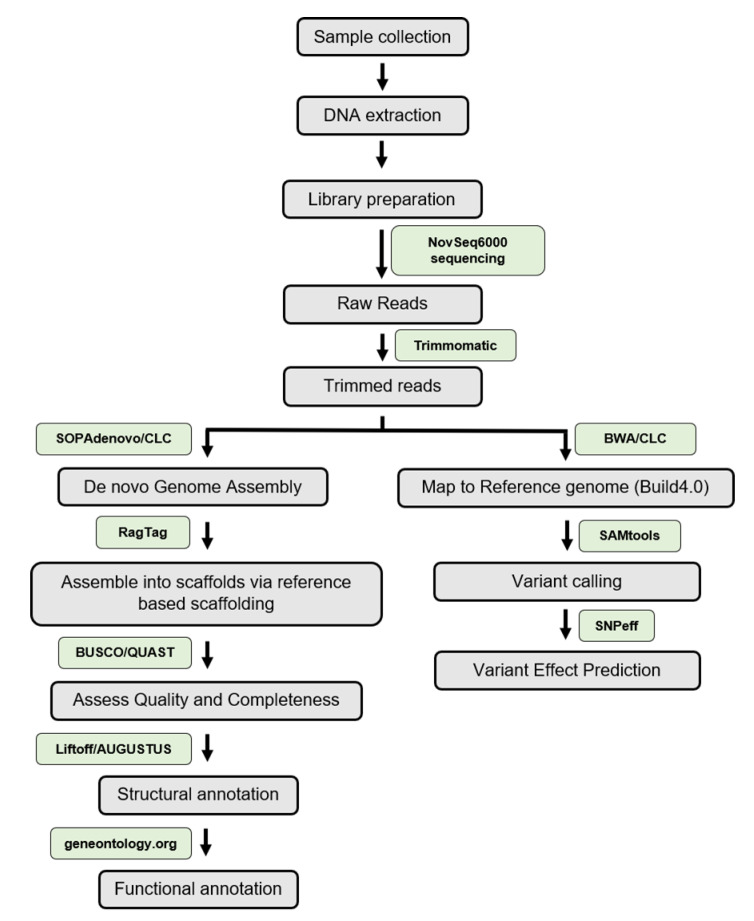
The workflow for reference guided assembly, annotation and variant calling used in this study. The major tools used have been highlighted in green boxes.

The PR genome was sequenced using the next generation short read sequencing platform (NovaSeq6000) with paired-end reads (151 bp read length) amounting to over 35 GB of total data (Table S1). The initial raw data was processed to remove adaptor sequences and low-quality bases, resulting in 30 GB of processed read data, which was then assembled de novo. From the de novo assembly, the putative mitochondrial, chloroplastic, and ribosomal DNA contigs were removed based on their homology to the known mitochondrial, chloroplastic, and ribosomal DNA sequences. The filtered de novo assembly was patched and scaffolded into 12 chromosomes using RagTag [38] based on the *S. lycopersicum* reference genome (Heinz1706; ITAG 4.0) (Table 1; Table S4). The pipeline used for generating draft PR genome assembly is outlined in Figure 3. The final PR assembly contains 96,632 contigs, out of which 13 are chromosomes (chromosome 00 to 12) and the rest are unplaced contigs (Table 1). The size of the assembled genome (~753 Mb) was within the range of the estimated tomato genome size (900 MB).

We used the Benchmarking Universal Single-Copy Orthologs (BUSCO) database to assess the completeness of the PR genome assembly [41,55,56]. Out of the 5950 BUSCOs searched in the solanales dataset, 97.7% (5816) were found to be completely present with only 115 missing, denoting a nearly complete genome assembly (Table S5). The quality of the assembly was further analyzed using QUAST (Table 2). The largest chromosome was of length 84,547,471 and the N50 for the PR genome assembly was 60,396,827, both comparable to the tomato reference assembly (Table 2). The unplaced contigs were kept as such in the final assembly (total size 35,061,362 bp) (Table S4 and Table 2). The final assembly was packaged according to PANsol guidelines and therefore named PR_v1.0.

### 3.3. Genome-Wide Analysis of Variants

**Figure 4:**
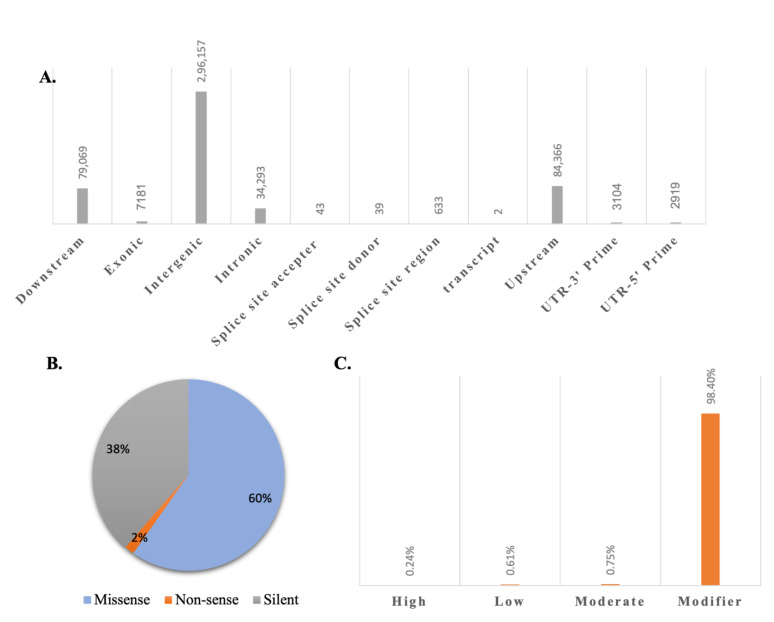
Number of variants in *S. lycopersicum* cv. Pusa Ruby genome classified (**A**) based on genomic region in which they are present, (**B**) by functional class of effects (in percent), (**C**) by impact (in percent). Variants can be divided into 11 classes based on position in the genome. These include the following mutations along with their contribution in the Pusa ruby genome: 1. downstream of the gene (15.57%), 2. in the exonic region (1.41%), 3. in the intergenic region (58.32%), 4. in the intronic region (6.75%), 5. in the splice acceptor site (0.008%), 6. in the splice donor site (0.007%), 7. in the splice site (0.12%), 8. mutation hits a transcript (0.0003%), 9. Upstream of a gene (16.6%), 10. variant hits 3′ prime untranslated region (0.61%), and 11. variant hits 5′ prime UTR (0.57%). Based on functional class, the mutations can be missense, non-sense, or silent, the majority being missense (60%) here in the Pusa Ruby genome. (**C**) Based on impact, the variants can have a high, low, moderate, or no effect. The variants with no effect are defined as modifier here, which also account for the majority (98.4%).

The paired-end reads for PR were mapped to the SL4.0 reference genome assembly (build SL4.0; https://solgenomics.net/ftp/tomato_genome/Heinz1706/assembly/, accessed on 22 April 2022) to call small variants as well as large structural variants. These variants included single nucleotide polymorphisms (SNPs), insertion deletions (InDels), multiple nucleotide variations (MNPs), and large structural variants. Breakpoints were also analyzed in the PR genome. A total of 342,963 variants were found in the PR genome as well as over 68,959 breakpoints on 12 PR chromosomes. Chromosome 4 has the maximum number of breakpoints (13,861), while chromosome 2 has the minimum number of breakpoints (Table S6). About 1720 larger structural variants were found in the PR genome, of which 105 were deletions, 365 were insertions, 21 were inversions, 1145 were replacements, and the rest were complex genetic variations. Additionally, 8533 variants were MNPs and 217,001 SNPs. We found 84,377 insertions and 31,670 deletions in the PR genome and 1382 variations were of mixed type. Over one hundred and seventy-four gene locations have more than one type of alleles. Out of all these variants, 1232 are of high impact, 3104 are of low impact, 3781 are of moderate impact, and 499,689 are modifier mutations. According to functional class, PR has 3532 missense (59.783%), 95 non-sense (1.608%), and 2281 silent (38.609%), with the missense to silent ratio being 1.5484. Most of the variants were in intergenic regions (296,157; 58.226%) (Figure 4; Table S7). Pusa Ruby also has 224 5′ UTR premature start codon gain variants and 110 stop gained variants. Transitions are the dominant type of mutations (122,740), with the transition to transversion ratio being 1.3043 (122,740/94,106) (Table S7).

### 3.4. Structural and Functional Annotation of Pusa Ruby Genome

We used liftoff to annotate the genes present in the PR genome assembly. A total of 34,075 protein coding genes were annotated, which is close to the number of genes in the reference genome (Heinz1706; 34,655). In the assembled PR genome, as mentioned before, 97.7% single copy genes were found (5813 of 5950) suggesting high quality. Of these 5816 single copy genes, a total of 5713 genes were completely single copy, while 103 were found to be duplicated (Table S5). Gene ontology terms were also assigned to the annotated sequences. Out of the 34,075 protein coding genes, 20,957 were uniquely mapped to known genes and were assigned GO terms and a panther class, while 13,118 remained unmapped (Files S3 and S4). For the molecular function GO category, most of the proteins had organocyclic and heterocyclic compound binding function and protein binding function, especially peroxisome targeting sequence binding (GO:0000268) and phospholipase activity (GO:000462; GO:0005515; GO:1901363; GO:0097159). Most of the annotated proteins were involved in cellular physiological processes including cellular communication. This was also confirmed in the overrepresentation test, as it was found that for the biological processes (BP) GO category, the proteins involved in cellular physiological processes for growth maintenance were at the maximum. For the cellular components GO category, proteins in the anatomical structure of cells were at the maximum (GO:0110165) (Files S3–S7). Moreover, the majority of the annotated proteins belonged to the class protein modifying enzymes (PC00260) (Figure S1).

### 3.5. Whole Genome Comparison of Pusa Ruby with Fla.8924, M82 and Heinz1706

Translocations, inversions, breakpoints, relocations, insertions, InDels, and SNPs were calculated using the DNAdiff tool via the alignment of PR with Heinz1706, M82 and Fla.8924 (Table 3). Heinz1706 is the variety on which the *S. lycopersicum* reference assembly and annotation (the latest being Build SL4.0 and annotation ITAG4.0) is based, while M82 is a processing cultivar frequently used for research purpose because of a rich genetic resource. Fla.8924 is a superior breeding line which is large-fruited and fresh market type, originally developed for field production in Florida [57]. The cultivars, M82 and Fla.8924, were used for the production of a pan-SV (structural variants) analysis for tomato in a study by [58].

The PR genome assembly (PR_v1.0) is the most similar to *S. lycopersicum* cv. Heinz1706 as it is guided by the Heinz1706-based reference assembly. Since only chromosomes and not unplaced contigs were used in this comparison, the PR genome assembly was shorter than the other counterparts. Even though the PR_v1.0 is shorter and has more Ns overall than Heinz1706, 96.12% of this assembly can be aligned with Heinz1706. The percent alignment is 94.58% between PR_v1.0 and Fla.8924, and 94.77% between PR_v1.0 and M82. The number of unaligned bases of PR_v1.0 with Heinz1706 (3.88%) is fewer than M82 (5.23%) and Fla.8924 (5.42%). This is also supported by the number of one-to-one alignment blocks between PR and Heinz1706 (232,149), Fla.8924 (216,975), and M82 (224,643). These one-to-one alignment blocks are a subset of M-to-M (many-to-many) mapping blocks of alignment of PR_v1.0 to Heinz1706, Fla.8924, and M82. The sum of length of these one-to-one alignment blocks is 682 Mb for Heinz1706, 665 Mb for Fla.8924, and 666 Mb for M82. The number of breakpoints between PR and Heinz1706 were comparable to Fla.8924 and M82, but relocations, translocations, inversions, average insertions, sum of tandem insertions, SNPs, and indel were quite lower than Fla.8924 and M82 (Table 3). This is consistent with the alignment results suggesting the order of similarity of PR genome with Heinz1706 (%) compared to Fla.8924 (%).

### 3.6. Comparative Evolutionary Genomics with Other Solanaceae Crops

**Figure 5:**
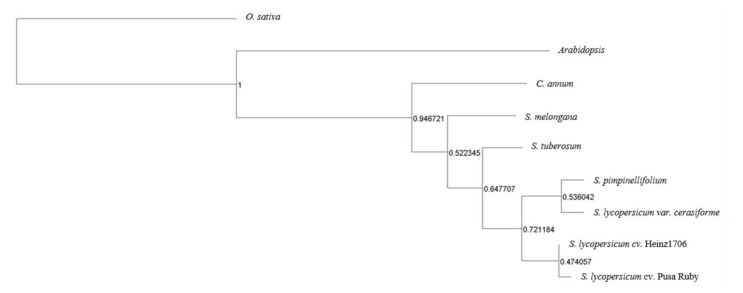
Phylogenetic tree depicting relationship between *S. lycopersicum* cv. Pusa Ruby and related Solanaceae species based on orthologous groups, and with rice and *Arabidopsis* as outgroups. The tree was generated using Orthofinder [51] 3.6 Allele mining of genes involved in Regeneration Efficiency.

Among the *S. lycopersicum* cv. Pusa Ruby and other related Solanaceae (*Capsicum annum, S. lycopersicum, S. melongana, S. lycoeprsicum var. cerasiforme, S. pimpinellifolium* and *S. tuberosum*), along with two outgroups (*Arabidopsis thaliana* and rice), 22,745 orthogroups were found. The maximum number of overlapping orthogroups of PR were found with *S. lycopersicum* Heinz1706 (31,070.0) and minimum with rice (17502.0), as expected. Other than these expected values, PR shared more orthologous groups with *S. lycopersicum* var*. cerasiforme* (26,129) than *S. pimpinellifolium* (25,797). Pusa Ruby shares common orthologous groups with other solanaceae species in the following order, *S. tuberosum, S. melongana,* and *C. annum* (Table S8). Further phylogenetic analysis confirmed the same (Figure 5).

### 3.7. Pusa Ruby Has Substantially High Regeneration and Transformation Efficiencies

**Figure 6:**
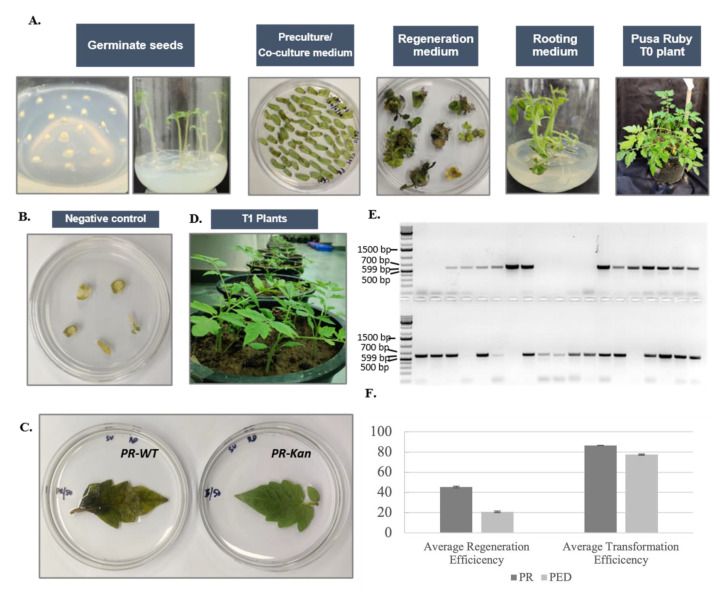
(**A**) Process of *Agrobacterium tumefaciens* mediated transformation of *S. lycopersium* var. Pusa Ruby and Pusa Early Dwarf. The image here represents Pusa Ruby only. (**B**) Explants not treated by *Agrobacterium* culture treated as control. (**C**) T1 transgenic plants growing in a greenhouse facility. (**E**) PCR and (**D**) kanamycin assay was used to select tranformants. Primers specific for kanamycin gene (nptII) were used for PCR that resulted in 599 bp long fragment. The PCR shown here is for one batch of plants with first two wells (1 and 2 in stack 1) with negative control wild type plants and 1 kb+ DNA ladder by Fisher scientific. (**F**) Regeneration and transformation efficiencies of Pusa Ruby (PR) and Pusa Early Dwarf (PED).

We chose Pusa Early Dwarf (PED) to compare the regeneration potential of PR. The regeneration efficiency for PR was found to be 45.5 ± 0.8, in comparison to PED which was 20.6 ± 0.8. The shoots regenerated from PR were especially efficient in rooting, as the varieties recalcitrant for regeneration in tomato, such as PED, have difficulty in rooting. However, the transformation efficiency for the regenerated plants was considerable (above 77%) for both varieties. Nevertheless, the transformation efficiency was higher for PR (86.5 ± 0.25%) than PED (77.4 ± 0.6%) in regenerated plantlets (Figure 6).

### 3.8. Allele Mining of Genes Involved in Regeneration Efficiency

Pusa Ruby is particularly amenable to efficient regeneration and transformation, as we described in the current study. Therefore, we analyzed the alleles for genes putatively involved in the process of acquiring totipotency and regeneration present in the PR genome (Table 4). Most of the genes contained reference type alleles in the intragenic region except *SOMATIC EMBRYOGENESIS RECEPTOR LIKE KINASE* (4), *BABY BOOM* (1), *cytokinin type-B ARRs* (1), *AGAMOUS-LIKE15* (50), *SlIAA9* (18), and *SlDOF9* (1) [59]. Using SNPeff, the identified variants could be classified as intergenic variants, genic variants, upstream gene variants, downstream gene variants, and intron variants. The variants could further be classified into in-frame InDels, frameshift variants, missense variants, synonymous variants, splice region variants, and start lost or stop gained variants. A total of 381 variants were found in 13 regeneration-related genes in Pusa Ruby. The maximum allele frequency was found to be associated with *SlIAA9* (271), and minimum with *LEAFY COTYLEDON1 (LEC1)* (null). Out of these variants, only one variant in *AGAMOUS-LIKE15* gene was found to be of high impact stop gained type.

### 3.9. Characterization of Variants Associated with Fruit Quality-Related and Other Domestication-Related Genes

We selected the most imperative representative genes involved in fruit quality in tomato for allele mining in PR (Table 5). About seven hundred and seventy-five variants were identified in the selected genes, out of which the majority were intergenic variants (614), and only three variants were missense, stop lost, and frame-shift type, one in each category. Most of these genes have reference-type alleles in the genic region, except *Chalcone isomerase (CHI), COMPOUND INFLORESCENCE(S), HIGH-PIGMENT1 (HP1)* and *2 (HP2), JOINTLESS (J1), OVATE (O),* and *RIPENING INHIBITOR (RIN).* JOINTLESS (JI), the gene involved in controlling the number of fruits or flowers by the induction of an abscission layer, has the maximum number of intragenic variants (62) while all others have a single intragenic variant on average. An *S-Rnase* (Solyc01g055200) that controls self-incompatibility in tomatoes has a maximum number of associated variants (409) in PR. Here, only two variants were found to be of high impact; one in the *O gene* that controls fruit shape, and the other in *HIGH PIGMENT 2* (Table 5).

## 4. Discussion

Access to high-quality crop genomes is of the utmost importance, considering the useful information gene model annotations can provide for functional and evolutionary genomics. The recent developments in plant whole genome sequencing have generated a vast amount of data, revealing variations in different genotypes within species. More than 250 important plant species have been resequenced at the whole genome level, such as Arabidopsis [91], rice [92], maize [93], and cotton [94]. Multiple tomato accessions have also been sequenced [6,95,96]. Here, we provide the genome of a principal tomato cultivar, PR, used especially for research because of its high regenerative potential, to reveal the genetic architecture at species level.

In this study, we deployed illumina-based NovaSeq6000 paired end (PE) short reads to sequence the whole genome of PR, generating 30 Gb data from single end. The PR genome size was estimated to be 723 Mb approximately, which is near to the assembled genome size of the *S. lycopersicum* cv. Heinz1706 (773 Mb) and within the range of estimated genome size of the tomato (950 Mb). The percentage error and duplication due to PCR were found to be low. The genome homozygosity and unique haploid sequence content was found to be high, making genome assembly facile.

The reads generated here were assembled into a draft genome using reference guided scaffolding. The de novo assembly generated using short PE reads w scaffolded into chromosomes using RagTag scaffold, a reference guided bioinformatic tool [38]. This draft assembly was named PR_v1.0 and packaged according to PanSol guidelines and the reference SL Build. The draft assembly generated thereof was assessed to be of comparable quality to the reference SL Build 4.0, with N50 equalling 60,396,827 and total contig count 96,632 (Table 2). The BUSCO analysis suggested 97.7% completeness of the assembly generated here. However, the size of genome assembly, the unplaced contig count, the number of N’s per KB (3686.50), and the number of unplaced base pair of PR_v1.0 is high and needs improvement, as well as gives scope for the identification of novel genes (Table 1 and Table 2).

The reference-guided assembly of PR identified 342,963 small variants and 2686 larger structural variants. Out of these, 63.27% account for SNPs and only 33.83% are insertion deletion mutations, with only 2.5% (8533) being multiple nucleotide variations, with 98.40% being modifier mutations only with no impact. The largest proportion of variants were in chromosome 12 (61,670), followed by chromosome 4 (35,393), and chromosome 2 (34,579). These results are consistent with previous findings [95]. Only 0.243% mutations were found to be of high impact, two of which were found in fruit quality-related genes and one in regeneration-related genes. These mutations may result in higher totipotency or regeneration response of PR. However, more research is required based on expression data in callus and other regeneration stages in PR cultivar to confirm the results to correlate regeneration response and SNPs. The mutation in ovate results in a near-perfect round shape, although with ribs, which is rare [97].

DNAdiff was used to calculate differences between two genomes. The way DNAdiff determines these differences is as follows. SNPs are the total number of single nucleotide polymorphisms. In an alignment, the count of non-maximal end points are breakpoints. A scaffold composed of contigs is followed by long stretches on N’s or gaps, which will not align. Thus, there will be one-to-one blocks which are aligning with breakpoints ending in the gap region. Relocations are defined when one-to-one alignments are on the same sequence but not in the same order or not consecutive. DNAdiff counts the ends of these relocations. Inversions are one-to-one count for the endpoints present in the same sequence which are adjacent but inverted. The translocations, in contrast, are counted for the endpoints of one-to-one alignments present on different sequences. Big sequences of more than 60 bp length that break the alignment and are non-gap regions are designated as insertions here. Insertion sum row is the count of total length of these insertions. Disruptions of less than 60 bp length are considered indels. The adjacent one-to-one alignments and duplications that follow the definition of insertion are tandem insertions here, and the tandem insertion row represents their total length. We used the default parameters in NUCmer, and therefore the alignment for PR refers to one-to-one and one-to-many, but for M82, Heinz1706, and Fla.8924, one-to-one alignments only are taken into account and this why there are two columns for the number of aligned and unaligned bases for each query-reference pair. It must be noted that the unplaced contigs were not taken into account for the analysis here, and therefore, the variant analysis results are different than BWA mapping. Pusa Ruby genome assembly is the most similar to Heinz1706 as expected, since it was based on the Heinz1706-based reference assembly. This is one of the drawbacks of reference-guided assembly. Although it is a fast and reliable method of generating a genome assembly, there is always a bias towards the reference.

Plant regeneration in tissue culture practices, especially in the case of *Agrobacterium* mediated transformation, is manipulated by the controlled supplementation of plant growth regulators. Solanaceae are particularly amenable to *Agrobacterium* mediated transformation, being a natural host to *Agrobacterium* sp., although the transformation percentage varies from species to species and among cultivars within a species. Currently, the choice of growth regulators, as well as their timing and duration of exposure, are central for increased efficiency. They are usually defined empirically for each species or even each genotype. Currently, the underlying molecular mechanisms are obtaining more attention, with stress playing a central role [60,61,98,99].

Fruit quality is the major defining feature for commercial exploitation of tomato plants, rendering the alleles for fruit quality-related genes imperative. A majority of the fruit quality-related genes have also been instrumental in domestication sweeps in the Lycopersicon clade. These include genes involved in fruit size, number, shape, weight, carotenoid and other anti-oxidant content, and those involved in shelf life of tomato fruits. Allele mining of the fruit quality-related genes in PR revealed a mutation in the *Ovate* gene, which is responsible for fruit shape [100]. *Ovate* is an important locus that has played a role in the domestication of wild varieties [101]. Round fruits of PR are a result of this mutation, which as we observed, were more amenable to fruit cracking especially during adverse environmental conditions, which is economically unacceptable. The *ovate* locus plays a role in the transformation of round fruits into pear shape. The domesticated varieties generally have an *ovate* allele which results in a premature stop codon. Here, we observed a stop loss type of mutation, resulting in inhibition of change of fruit shape from round to pear. Further, the high-pigment 2 mutation found in PR visibly has no effect on the plant growth as is generally characterized for hp2 mutant tomato plants [102]. The data generated here can be used to mine additional novel alleles that can be used in the production of designer crops.

## 5. Conclusions

The natural variations in the coding as well as regulatory regions identified here can be utilized for both forward and reverse genetics for the betterment of tomato varieties, aiding the current genomic resources. The pipeline and codes for reference-guided assembly given here can also be used by the researcher directly for any crop.

## Figures and Tables

**Table 1:** Comparison of chromosome length of Heinz1706, which is used as reference genome assembly and Pusa Ruby genome assembled in the current study.

Chromosome	Chromosome Length (bp) Heinz1706	Chromosome Length (bp) Pusa Ruby	Number of Contigs from the Pusa Ruby De Novo Assembly Ordered and Oriented by RagTag
chr 1	90,863,682	84,547,471	5703
chr 2	53,473,368	49,336,188	33,805
chr 3	65,298,490	60,101,449	44,527
chr 4	64,459,972	60,396,827	41,165
chr 5	65,269,487	60,896,774	46,821
chr 6	47,258,699	43,868,379	30,453
chr 7	67,883,646	62,983,648	47,809
chr 8	63,995,357	59,251,915	45,563
chr 9	68,513,564	64,410,981	48,037
chr 10	64,792,705	59,814,738	46,567
chr 11	54,379,777	49,956,557	39,443
chr 12	66,688,036	60,856,427	47,439
Sum	772,876,783	716,421,354	

**Table 2:** QUAST statistics for the *S. lycopersicum* var. Heinz1706 reference genome and the *S. lycopersicum* cv. Pusa Ruby assembly by ragtag [38]. # - number.

Statistics without Reference	Heinz1706 Reference	PR
# contigs or scaffolds	13	96,632
# contigs or scaffolds (≥0 bp)	13	96,632
# contigs or scaffolds (≥1000 bp)	13	4568
# contigs (≥25,000 bp)	13	25
# contigs (≥50,000 bp)	13	15
Largest contig or scaffolds	90,863,682	84,547,471
Total length (≥0 bp)	782,520,033	753,700,435
Total length (≥1000 bp)	782,520,033	729,632,876
Total length (≥25,000 bp)	782,520,033	718,987,089
Total length (≥50,000 bp)	782,520,033	718,782,531
Total length	782,520,033	753,700,435
N50	65,269,487	60,396,827
N90	53,473,368	49,336,188
L50	6	6
L90	11	11
GC (%)	34.34	33.84

**Table 3:** Pairwise whole genome comparison of Pusa Ruby against Heinz1706, Fla1, and M82 computed by DNAdiff tool [50]. Pusa Ruby was the query while Heinz1706, Fla.8924, and M82 were the references in the NUCmer alignment and DNAdiff tool. Seqs: Sequences; Avg: average; SNPs: Single Nucleotide Polymorphisms; GSNPs: Single Nucleotide Polymorphisms(SNPs) bound by 20 exact base pair matches on either side; Indel: Insertion/deletions; GInDels: Insertions/deletions bound by exact match of 20 base pairs on either side; PR: Pusa Ruby.

	Heinz1706	PR	Fla.8924	PR	M82	PR
**Sequences**						
*Total Seqs*	13	13	13	13	13	13
*Aligned Seqs*	13 (100.00%)	13 (100.00%)	13 (100.00%)	13 (100.00%)	13 (100.00%)	13 (100.00%)
*Unaligned Seqs*	0 (0.00%)	0 (0.00%)	0 (0.00%)	0 (0.00%)	0 (0.00%)	0 (0.00%)
**Bases**						
*Total Bases*	782,520,033	718,639,073	796,004,315	718,639,073	792,934,937	718,639,073
*Aligned Bases*	726,051,384 (92.78%)	690,758,192 (96.12%)	715,955,700 (89.9437%)	679,673,622 (94.58%)	717,409,934 (90.48%)	681,068,925 (94.77%)
*Unaligned Bases*	56,468,649 (7.22%)	27,880,881 (3.88%)	80,048,615 (10.0563)	38,965,451 (5.42%)	75,525,003 (9.52%)	37,570,148 (5.23%)
**Alignments**						
*one-to-one*	232,149		216,975		224,643	
*Total Length (one-to-one)*	681,763,285	681,795,255	663,171,553	664,463,584	665,697,245	665,915,093
*Avg Length (one-to-one)*	2936.75	2936.89	3056.725	3062.67705	2964.6299	2902.57
*Avg Identity (one-to-one)*	99.84		99.5656		99.5777	
*M-to-M*	399,293		393,605		401,181	
*Total Length (M-to-M)*	771,135,016	771,128,110	781,072,565	783,869,191	774,217,622	774,798,581
*Avg Length (M-to-M)*	1931.25	1931.23	1985.6105	1992.7126	1930.32895	1931.7748
*Avg Identity (M-to-M)*	99.15		98.5888		98.66545	
**Feature Estimates**						
*Breakpoints*	798,577	798,553	787,208	787,189	802,352	802,339
*Relocations*	26,309	17,453	28,508	27,573	28,697	27,028
*Translocations*	7328	7221	16,357	14,667	12,041	11,130
*Inversions*	714	718	3173	2644	2422	2112
*Insertions*	242,155	319,388	235,641	323,561	247,574	323,754
*Insertion Sum*	130,014,304	49,843,243	162,162,183	80,350,739	156,691,957	70,683,091
*Insertion Avg*	536.91	156.06	688.5165	248.3442	633.373	218.5611
*Tandem Ins*	321	42	374	61	400	110
*Sum of Tandem Ins*	92,910	2859	59,361	4491	76,900	9232
*Tandem Ins Avg*	289.44	68.07	157.92195	72.5741	192.08725	83.38
**SNPs**						
*Total SNPs*	335,439		786,423		1,452,890	
*Total GSNPs*	167,484		266,642		734,607	
*Total InDels*	356,419		1,572,447		880,972	
*Total GInDels*	94,663		54,287		56,923	

**Table 4:** Allele mining of genes involved in regeneration process or totipotency and cell fate in *S. lycopersicum* cv. Pusa Ruby.

Gene	Arabidopsis Homologue	GENE ID	ITAG 4.0 Positions	Number of Variants in PR	High Impact Variant	Reference
*SOMATIC EMBRYOGENESIS RECEPTOR LIKE KINASE*	NA	*Solyc04g072570.3*	57484887…57491796	13	N	[59,60,61,62]
*CLAVATA 1*	NA	*Solyc11g071380*	52945095…52945649	3	N	[59,60,61,62,63]
*WUSCHEL*	NA	*Solyc02g083950.3.1*	45191157…45192582	11	N	
*SHOOT MERISTEMLESS*	NA	*Solyc02g081120.4*	43175431…43179388	2	N	
*BABY BOOM*	NA	*Solyc11g008560.2*	2782134…2787131	4	N	[59,64,65]
*Cytokinin type-B ARABIDOPSIS RESPONSE REGULATORs*	At4g31920	*Solyc12g010330.2*	3431027…3436636	5	N	[66,67]
*LEAFY COTYLEDON1 (LEC1)*	At1g21970	*Solyc04g015060.3*	5289310…5292118	0	N	[68,69,70]
*AGAMOUS-LIKE15*	NA	*Solyc01g087990.3*	75071660…75077872	51	Y; c.562C > T; p.Arg188 *; STOP GAINED	
*RETICULON-LIKE3 (RTNLB3)*	AT1G64090	*Solyc01g095200.3.1; Solyc03g007740.3.1*	78807148…78810378; 2297766…2300313	0; 1	N	[71]
*RETICULON-LIKE8 (RTNLB8)*	AT3G10260	*Solyc12g006290.2.1*	809990…814489	2	N	[71]
*SlIAA9*	NA	*Solyc04g076850.3*	59750087…59755552	271	N	[72]
*SlDOF9*	NA	*Solyc02g090220.3*	49886648…49887678	19	N	[73]

**Table 5:** Allele mining of genes involved in fruit quality regulation in *S. lycopersicum* cv. Pusa Ruby.

S.No.	Gene	Gene ID	Gene Function	Position in ITAG 4.0	Number of Variants in PR	High Impact Variant	Reference
1	AUXIN RESPONSE FACTOR 19 (ARF19)	*Solyc07g042260.4*	*Negative regulator of fruit set*	55179909…55187268	4	N	[74]
2	CHALCONE ISOMERASE (CHI)	*Solyc06g084260.3*	*Contributes in flavonoid biosynthesis*	47032434…47037684	1	N	[75]
3	COMPOUND INFLORESCENCE (S)	*Solyc02g077390.2*	*Affects number of flower/fruits per inflorescence*	40324933…40326867	22	N	[76]
4	FASCIATED (FAS)	*Solyc11g071380.1*	*Controls number of carpels/locules in the fruit*	52945095…52945649	2	N	[77]
5	FRUIT WEIGHT 2.2 (FW2.2)	*Solyc02g090730.3*	*Quantitative variation of fruit size*	50292691…50293481	3	N	[78]
6	GOLDEN2-LIKE (GLK2)	*Solyc10g008160.3*	*Controls chloroplast development in fruits*	2169302…2174039	0	N	[79]
7	HIGH-PIGMENT 1 (HP1)	*Solyc02g021650*	*Controls anthocyanin accumulation in fruits*	21799951…21820357	23	N	[80]
8	HIGH-PIGMENT 2 (HP2)	*Solyc01g056340*	*Controls anthocyanin accumulation in fruits*	46851339…46871997	127	Y; FRAME SHIFT; c.1503dupC; p.His503fs	[80]
9	JOINTLESS1 (J1)	*Solyc11g010570*	*Controls abscission zone formation in pedicels*	3671232…3676350	148	N	[81]
10	LIN5	*Solyc09g010080*	*Extracellular invertase expressed in the fruit ovary*	3508156…3512282	1	N	[82]
11	LYCOPENE β CYCLASE (Cyc-B)	*Solyc06g074240*	*Conversion of lycopene into B-carotene*	43562526…43564022	0	N	[83]
12	NON RIPENING (NOR)	*Solyc10g006880*	*Controls the initiation of the normal fruit ripe program*	1191950…1194846	3	N	[84]
13	OVATE (O)	*Solyc02g085500*	*Regulates fruit shape*	46380291…46382023	3	Y; STOP LOST; c.856T > G; p.Ter286Gluext *	[85]
14	RIPENING INHIBITOR (RIN)	*Solyc05g012020*	*Controls ripening-related ethylene biosynthesis*	5302348…5308593	9	N	[84]
15	SINGLE FLOWER TRUSS (SFT)	*Solyc03g063100*	*Flowering inducer*	29218239…29222055	9	N	[86]
16	S-RNAse	*Solyc01g055200*	*Controls Self-incompatibility*	44944259…44945021	409	N	[87]
17	Pectate Lyase (PL)	*Solyc03g111690*	*Control pectin degradation in cell wall*	56752862…56755013	9	N	[88]
18	Plygalacturanase (POLYGAL)	*Solyc07g056290*	*Cell wall degrading enzyme*	64064076…64067658	2	N	[89]
19	9-cis-epoxycarotenoid dioxygenase (NCED)	*Solyc07g056570*	*Involved in ABA biosysnthesis*	64278530…64280347	6	N	[90]
20	Β-1-3-galactosyl transferase (GAL1)	*Solyc07g052320*	*Regulation of fruit galactose levels*	60727212…60731146	1	N	

## Data Availability

The raw data has been submitted to NCBI SRA under with accession ID PRJNA907318; https://www.ncbi.nlm.nih.gov/sra/PRJNA907318.

## Supplementary Materials

The following supporting information can be downloaded at: https://www.mdpi.com/article/10.3390/genes14030570/s1, Figure S1. The bar charts depicting top GO terms for molecular function, cellular component, and biological processes GO categories.; Table S1. Illumina library information and sequencing statistics before and after Trimmomatic processing and read alignment statistics.; Table S2. Accession IDs of organellar genome used to filter organellar genome sequences from the Pusa Ruby Genome assembly.; Table S3. Oligos used in this study for the identification of transgenics. Table S4. Statistics of ragtag scaffolding of Pusa Ruby *de novo* assembly.; Table S5. Short summary of results for BUSCOs for genome assembly validation of *S. lycopersicum* cv. Pusa Ruby out of the total 5950 BUSCO groups searched in the solanales lineage dataset (solanales_odb10).; Table S6. Variant rate and number of breakpoints in *S. lycopersicum* cv. Pusa Ruby genome.; Table S7. Number of effects of genomic variants in Pusa Ruby by type.; Table S8. Number of orthologous groups identified by OrthoFinder 2 between respective species/varieties.; File S1. Code Availaibility.; File S2a. Characteristics of *S. lycopersicum* cv. Pusa Ruby genome as calculated by the commandline version of GenomeScope version 1.0 using the short reads generated in our study and a k-mer size of 25 (http://qb.cshl.edu/genomescope/, accessed on 22 April 2022). Here, genome heterozygosity, genome haploid size, repeat content, unique length, model fit and read error rate have been dissected. The formula used by the Genomescope software, parameters used, significance codes, residual standard error, number of iterations to convergence, and achieved convergence tolerance have been described.; File S2b. Characteristics of *S. lycopersicum* cv. Pusa Ruby genome as calculated by the commandline version of GenomeScope version 1.0 using the short reads generated in our study and a k-mer size of 25 (http://qb.cshl.edu/genomescope/, accessed on 22 April 2022). Here, genome heterozygosity, genome haploid size, repeat content, unique length, model fit and read error rate have been dissected. The formula used by the Genomescope software, parameters used, significance codes, residual standard error, number of iterations to convergence, and achieved convergence tolerance have been described. File S3. Gene list of mapped genes with known functions. PR mapped IDs to the ensembl IDs are given with gene name and gene symbol if available. Panther family and class assigned to each protein are also given.; File S4. Gene ontology terms assigned to protein coding Pusa Ruby genes by the online tool http://geneontology.org.; File S5. Panther overrepresentation test for Cellular Components GO category for Pusa Ruby (PR) protein coding genes. GO database used: GO Ontology database DOI: 10.5281/zenodo.6399963 Released 2022-03-22.; File S6. Panther overrepresentation test for Biological Processes GO category for Pusa Ruby (PR) protein coding genes. GO database used: GO Ontology database DOI: 10.5281/zenodo.6399963 Released 2022-03-22.; File S7. Panther overrepresentation test for Molecular Function GO category for Pusa Ruby (PR) protein coding genes. GO database used: GO Ontology database DOI: 10.5281/zenodo.6399963 Released 2022-03-22.
