# Supplementary material for: Reference Guided De Novo Genome Assembly of Transformation Pliable Solanum lycopersicum cv. Pusa Ruby
Source: Genes (Basel). 2023 Feb 24;14(3):570. doi: 10.3390/genes14030570 (PMC10047940; doi:10.3390/genes14030570)
Supplement: Supplementary file 1 [file genes-14-00570-s001.zip › File S1.pdf]

Code availability

JELLYFISH: jellyfish count -C -m 21 -s 1000000000 -t 10 *.fastq -o reads.jf ; jellyfish histo -t 10 reads.jf > reads.histo

GenomeScope: Rscript genomescope.R histogram_file k-mer_length read_length output_dir [kmer_max] [verbose]

BLAST: makeblastdb -in <input mitochondria/chloroplast/rDNA.fasta file> -parse_seqids -dbtype nucl -out <output file (blast database)>; blastn -db <blast database name> -query <de novo assembly fasta file> -out <output file> -outfmt "6 qseqid sseqid pident length mismatch gapopen qstart qend sstart send evalue bitscore qcovs"

Filtering selected chloroplastic, mitochondrial and rDNA contigs: python filter_fasta.py <text file with list of contigs to be removed> > <filtered de novo genome assembly>

RepeatMasker [28], version 4.1.0, parameters: -e ncbi -pa 16 -s -species tomato -noisy -excln -a -inv -xsmall; databases: Dfam [88] version 3.1 and Repbase [89] v20170127

RagTag: ragtag scaffold <reference genome fasta file> <de novo assembly contigs>

BUSCO (Manni, Berkeley et al. 2021), version 5.3.2; lineage dataset: solanales_odb10 (eukaryota, 2020-08-05); parameters: -f -i < Pusa Ruby assembly fasta file (PR_v1.0.fasta)> -m genome --lineage_dataset solanales_odb10

QUAST: Parameters for analysis without reference python quast.py -o <output directory> -e --no-snps <Pusa Ruby genome assembly fasta file with unplaced contigs>; Parameters for analysis with reference genome: quast.py -o <output directory> -r <reference genome (Build SL4.0)> -g <ITAG4.0_gene_models.gff> -s -e --circos -f --eu -b <Pusa Ruby assembly fasta file (PR_v1.0.fasta)>

Augustus (Stanke, Steinkamp et al. 2004), version 2.5.5, command: augustus --strand=both --genemodel=partial --gff3=on --species=tomato <PR genome fasta file>

for mummerplot: MUMmer [77], version 4.0.0beta2, command: nucmer, (default); delta-filter, version (parameters: -m -l 5000); mummerplot, version 3.5 (parameters: --color --png --large --layout); sequence similarity via dnadiff [31], version 1.3 (default)

NUCmer, MUMmer version 4.0.0rc1 (Marçais, Delcher et al. 2018), default parameters; command: nucmer -p <ouput file prefix> < reference assembly fasta file> <Pusa Ruby assembly fasta file (PR_v1.0.fasta)>

Dnadiff, MUMmer version 4.0.0rc1 (Marçais, Delcher et al. 2018), command: dnadiff -d <delta file generated by NUCmer>

all2vcf script for the extraction of vcf file from dnadiff output: mummer –snps <snps file generated by dnadiff> --reference <reference genome file> --input-header > <output file>

Mapping of Illumina short reads via BWA (Li and Durbin 2009), version 0.7.17 (default) and sorting of bam file via samtools, version 1.9, command samtools view, (parameters: -h -b -S), samtools sort, (default); samtools index, (default).

Liftoff (Shumate and Salzberg 2021). command : liftoff -g ITAG4.0_gene_models.gff -o <output file prefix> -u <unmapped_liftoff> -flank 0.2 -infer_genes -copies <Pusa Ruby assembly fasta file (PR_v1.0.fasta)> <reference genome fasta file>

gffread (Pertea and Pertea 2020), version 0.11.8, command for cds and transcript extraction: gffread -g < Pusa Ruby assembly fasta file (PR_v1.0.fasta)> <Pusa Ruby .gff file> --sort-alpha -F -Z -L -x -y -o PR2_gffread -w PR_transcripts2.fa

orthofinder (Emms and Kelly 2019) version 2.2.7. Default parameters used. orthofinder -f <directory with protein sequences of target genomes>

EDTA (Ou, Su et al. 2019) version. EDTA.pl --genome "/mstore/compute/users/humira/ragtag_output/ragtag.scaffold-Copy.fasta" --cds ITAG4.0_CDS.fasta --sensitive 1 --anno 1 evaluate 1

bwa mem -p -C <assembly fasta file> <barcoded linked reads fastq> | samtools sort -tBX -o <output bam file>
