# Supplementary material for: Reference Guided De Novo Genome Assembly of Transformation Pliable Solanum lycopersicum cv. Pusa Ruby
Source: Genes (Basel). 2023 Feb 24;14(3):570. doi: 10.3390/genes14030570 (PMC10047940; doi:10.3390/genes14030570)
Supplement: Supplementary file 1 [file genes-14-00570-s001.zip › Supplementary figures and tables.pdf]

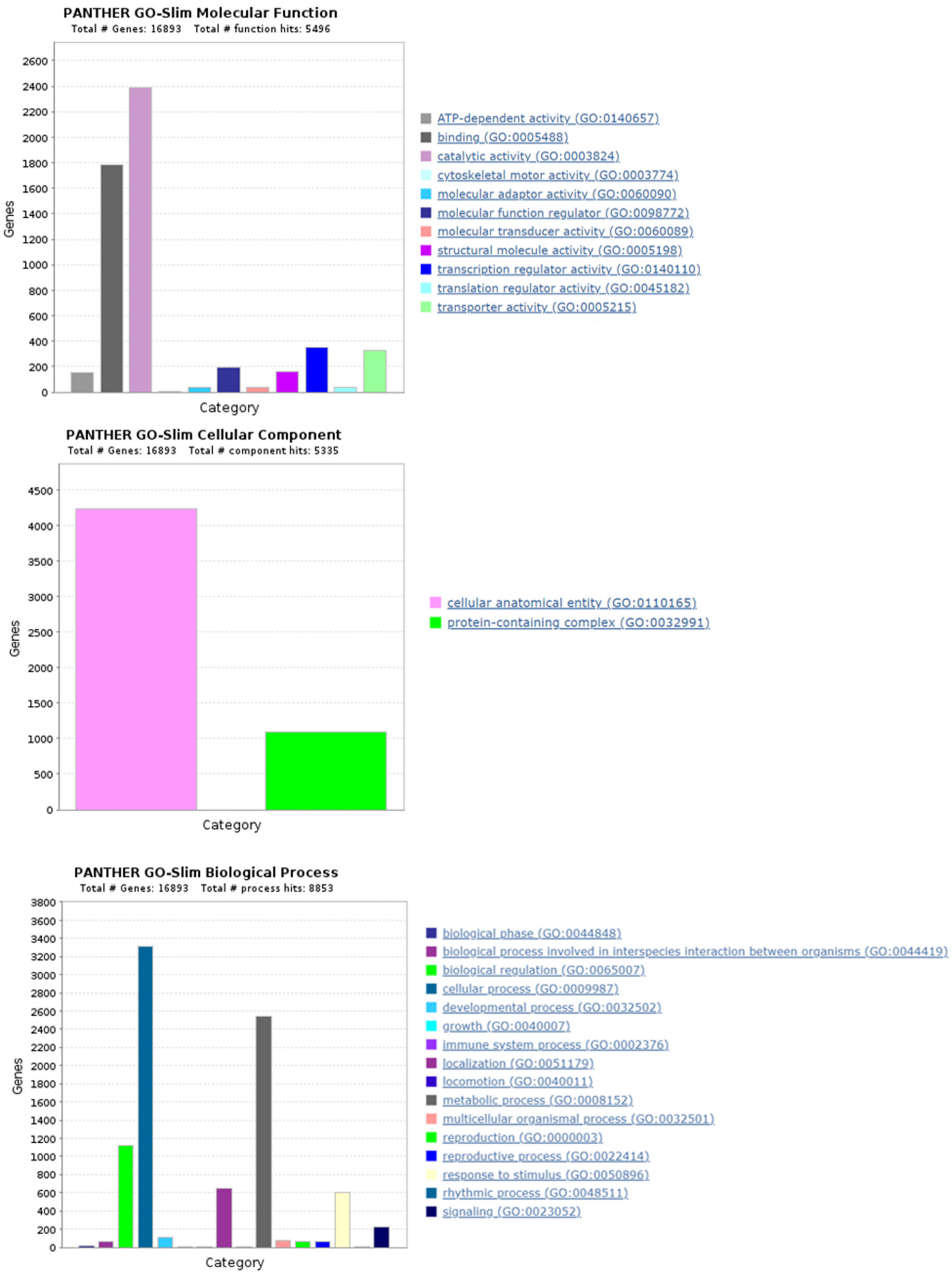

Figure S1. The bar charts depicting top GO terms for molecular function, cellular component, and biological processes GO categories.

Table S1. Illumina library information and sequencing statistics before and after Trimmomatic processing and read alignment statistics.

**Type**		Pair End
**Library name**		*PR1*
**Read length (bp)**	**Before**	151
	**After**	~136
**% bases >Q20**		99.92
**%bases >Q30**		98.35
**Reads**	**Before**	202457986
	**After**	202296504
**% GC**	**Before**	37
	**After**	36
**Mapped Reads (%)**		0.9989

Table S2. Accession IDs of organellar genome used to filter organellar genome sequences from the Pusa Ruby Genome assembly.

Organelle	GenBank accession IDs
Chloroplast	MN218076.1
	MN218077.1
	MN218078.1
	MN218079.1
	MN218091.1
	MN218088.1
	MN218089.1
	NC_039611.1
	NC_035724.1
	KX792501.2
	NC_041604.1
	MH283721.1
	NC_039605.1
	NC_039600.1
	NC_007898.3
	MN218081.1
	NC_039606.1
	NC_030207.1
	MT120858.1
	MN635796.1
	MN218090.1
	MT120855.1
	MT120856.1
	NC_050206.1
	MN218087.1
	NC_008096.2
Mitochondria	MT122954.1
	MT122955.1
	MT122966.1
	MT122969.1
	MT122973.1
	MT122974.1
	MT122977.1
	MT122988.1
	NC_050335.1
	MT122980.1
	MT122981.1
	MT122982.1
	MT122983.1
	MF989960.1
	MF989961.1

	NC_035963.1
	MT122970.1
	MT122971.1
	NC_050334.1
	MW122958.1
	MW122959.1
	MW122960.1
	MT122964.1
	MT122965.1
	MW122949.1
	MW122950.1
	MW122951.1
	MW122952.1
	MW122953.1
	MW122954.1
	MW122961.1
	MW122962.1
	MW122963.1
	MT122978.1
	MT122979.1
	MF989953.1
	MF989957.1
	MN114537.1
	MN114538.1
	MN114539.1
	MT122958.1
	MT122959.1.
rDNA	X55697.1
	AY366528.1
	AY366529.1
	KF156909.1
	KF156910.1
	KF156911.1
	KF156912.1
	KF156913.1
	KF156914.1
	KF156915.1
	KF156916.1
	KF156917.1
	KF156918.1
	KF156919.1
	KF156920.1
	KF156921.1
	KF156922.1

	KF603895.1
	KF603896.1
	X65489.1
	X82780.1
	AF464863.1
	AF464865.1
	AY366530.1
	AY366531.1
	AY875827.1

Table S3. Oligos used in this study for the various steps of genome editing. Abbreviations: sgRNA: single guide RNA.

Purpose	Oligo ID	Sequence	Tm	Amplicon size
**sgRNA**	gRNA3	AGTCCCCGGCTTCGGAGTGA	-	-
	gRNA4	AATCTACGTGGTGACCGATT	-	-
**Transgene analysis**	Cas9_F	CGCATCTCTCGGAACCTACC	58	957
	Cas9_R	CCTCCCCTCTCAGCCTTAGT	60	
**Mutant Screening**	SlPL_Sq_F2	GCCTCCTCTAATCCTCAACAAG	58	558
	SlPL_S1_R2	GTTATACACGGACCACCCGC	61	
	SlPL_Sq_F1	GGTTAGAGGAATCTCCGTAAAACA	56	1292
	SlPL_Sq_R1	TGTGTGTAGTCATTGTTCACCA	54	
**Off-target analysis**	g1-Off-PL1-F	GCGGTGGCTCAATATCCTCC	60	685
	g1-Off-PL1-R	GTTTTCATTCGAACCCTCCCC	59	
	g1-Off-PL2-F	TCTGGCGTTGTGGGAATTTTG	59	577
	g1-Off-PL2-R	AGCTGGTGGATTCTCCATTCTT	59	

Table S4. Statistics of ragtag scaffolding of Pusa Ruby *de novo* assembly.

**Parameter**	**Value**
Placed Sequences	270787
Placed base pairs	691561673
Unplaced sequences	96619
Unplaced base pair	35061362
Gap base pair	27077400
Gap Sequences	270774

Table S5. Short summary of results for BUSCOs for genome assembly validation of *S. lycopersicum cv. Pusa Ruby* out of the total 5950 BUSCO groups searched in the solanales lineage dataset (solanales_odb10).

**Type of BUSCO**	
Complete BUSCOs	58116
Complete and single-copy BUSCOs (S)	5713
Complete and duplicated BUSCOs	103
Fragmented BUSCOs	24
Missing BUSCOs	110

Table S6. Variant rate and number of breakpoints in *S. lycopersicum* cv. Pusa Ruby genome.

**Chromosome**	**Number of break points**	**Variants**	**Variant rate**
1	7,999	34,579	2,627
2	4,424	16,694	3,203
3	5,010	16,170	4,038
4	13,861	35,393	1,821
5	11,723	29,146	2,239
6	4,600	27,339	1,728
7	4,457	12,545	5,411
8	5,044	21,690	2,950
9	5,014	19,224	3,563
10	4,753	19,323	3,353
11	5,037	26,263	2,070
12	6,772	61,670	1,081

Table S7. Number of effects of genomic variants in Pusa Ruby by type.

Type	Count	Percent
3_prime_UTR_variant	3,104	0.61%
5_prime_UTR_premature_start_codon_gain_variant	224	0.04%
5_prime_UTR_variant	2,695	0.53%
conservative_inframe_deletion	13	0.00%
conservative_inframe_insertion	20	0.00%
disruptive_inframe_deletion	27	0.01%
disruptive_inframe_insertion	21	0.00%
downstream_gene_variant	79,069	15.55%
frameshift_variant	1,012	0.20%
initiator_codon_variant	2	0%
intergenic_region	2,96,157	58.23%
intragenic_variant	1	0%
intron_variant	34,905	6.86%
missense_variant	3,712	0.73%
non_coding_transcript_variant	1	0%
splice_acceptor_variant	43	0.01%
splice_donor_variant	39	0.01%
splice_region_variant	748	0.15%

start_lost	36	0.01%
start_retained_variant	2	0%
stop_gained	110	0.02%
stop_lost	40	0.01%
stop_retained_variant	6	0.00%
synonymous_variant	2,284	0.45%
upstream_gene_variant	84,366	16.59%

Table S8. Number of orthologous groups identified by OrthoFinder 2 between respective species/varieties.

	Arabidopsis	***C. annum***	***O. sativa***	***S. lycopersicum* cv. Heinz_1706**	***S. lycopersicum* var. *cerasiforme***	***S. melongana***	***S. pimpinellifolium***	***S. tuberosum***	***S. lycopersicum cv. Pusa Ruby***
Arabidopsis	0	30138	29628	31431	30847	31268	30818	29302	31050
C. annum	19728	0	18595	23358	24095	24255	24142	24457	22763
O. sativa	19917	20228	0	20841	20534	20745	20522	20030	20662
***S. lycopersicum* cv. Heinz_1706**	19522	22204	18616	0	27865	22380	27512	22689	31575
S. lycopersicum* var. *cerasiforme	19551	25066	18969	32642	0	26637	53861	26694	30182
S. melongana	19540	23614	18545	22900	24912	0	24868	23836	22406
S. pimpinellifolium	19656	25148	19137	32158	53446	26871	0	26920	29873
S. tuberosum	28430	34309	27404	32491	36191	34846	36813	0	31583

S. lycopersicum cv. Pusa Ruby	18216	20917	17502	31070	26129	21327	25797	21373	0
